# Late secondary urological reconstruction of separated ischiopagus twins with exstrophic bladder and urinary incontinence

**DOI:** 10.31744/einstein_journal/2018RC3887

**Published:** 2018-10-30

**Authors:** Antonio Macedo, Marcela Leal da Cruz

**Affiliations:** 1 Centro de Apoio à Criança com Anomalia Urológica, São Paulo, SP, Brazil;; Universidade Federal de São Paulo, São Paulo, SP, Brazil.; 2 Centro de Apoio à Criança com Anomalia Urológica, São Paulo, SP, Brazil.

**Keywords:** Urinary tract/surgery, Reconstruction, Urologic surgical procedures, Twins, conjoined, Sistema urinário/cirurgia, Reconstrução, Procedimentos cirúrgicos urológicos, Gêmeos unidos

## Abstract

We report a case of secondary urinary reconstruction of previously separated conjoined twins with exstrophic bladder and urinary incontinence. Patients were male and aged 13-year-old. Twin one had a history of failed enterocystoplasty that extruded and was visible like an exstrophic neobladder. He underwent a procedure to close bladder neck and reconfigure abdominal wall. After the procedure the patient developed a fistula that was treated, but it persisted and, for this reason, a catheterizable pouch was constructed and native bladder was discarded. Twin two required the immediately construction of catheterizable pouch using the Macedo’s technique. Currently, both patients are continent at 4 hour intervals. The mean follow-up was 8 months. Modern continent urinary diversion techniques offer new perspectives and hope for such complex population.

## RESUMO

Relata-se caso de reconstrução urinária em gêmeos siameses previamente separados com apresentação clínica de bexiga extrófica e incontinência urinária. Os dois pacientes eram do sexo masculino com idade de 13 anos. O primeiro gêmeo apresentava falha da enterocistoplastia com extrusão e visualização da neobexiga extrófica, tendo sido submetido ao fechamento do colo vesical e à reconfiguração da parede abdominal. Após o procedimento, o paciente desenvolveu fístula, que foi tratada, mas persistiu. Posteriormente, optamos por bolsa cateterizável, descartando a bexiga nativa. O segundo gêmeo foi submetido à construção imediata de bolsa cateterizável, por meio da técnica de Macedo. Atualmente, ambos os pacientes estão continentes em intervalos de 4 horas. O seguimento médio foi de 8 meses. As atuais técnicas de derivação urinária oferecem novas perspectivas e esperança para esta população complexa.

## INTRODUCTION

Conjoined twin is a rare condition with estimated incidence of 1 in 50,000 live births. This affection is more common in females with sex ratio of 3:1.^(^ ^1^ ^,^ ^2^ ^)^ Such patients are monozygotic and monochorionic twins. The incomplete separation of the inner cell mass around 13 to 15 days of gestation is responsible for this abnormality.^(^ ^3^ ^)^ Conjoined twins can be joined at different sites, and those classified as ischiopagus (joined by ischium) had higher risk of lower urinary tract complications.

Separation rarely involves urgent issues, and ideal time for separation is within the first year of life, as this period is adequate to enable the necessary investigations prior to the procedure.

To date, few reports have deal with long-term follow-up particularly concerning urological functioning and urinary continence. We report a case of secondary urinary reconstruction in previously separated conjoined twins with exstrophic bladder and who were aged 13 years.

## CASE REPORT

**Figure 1:**
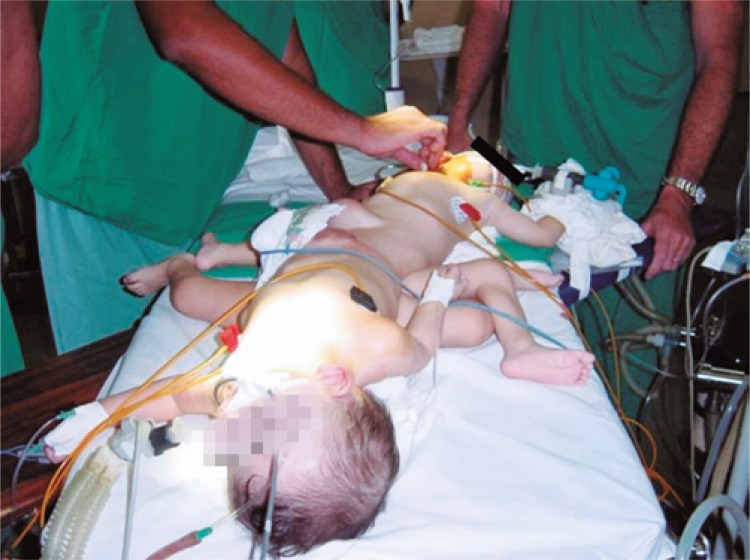
Male tetrapus twins joined from lower abdomen to perineum

These were male tetrapus twins weighing 3,900g and who were delivered by Caesarean section. They were face to face and joined from the lower abdomen to the perineum ( Figure 1 ). A third twin from different gestational sac was also born. Each conjoined twin had two normal kidneys and two ureters, but both presented classic bladder exstrophy-epispadia complex. In their perineum there were two separate penises with epispadia, one well developed hemiscrotum in each patient, and no evidence of testes in the undeveloped side.

When twins were 8 months of age, they underwent separation surgery. The large colon with dual blood supply was divided longitudinally. The pelvic organs were reviewed, and bladder and genital tracts were apportioned.

After their successful separation, twin 1 at age of 3.8 years underwent the first urological procedure that consisted of primary bladder exstrophy closure. He had bladder dehiscence, therefore, subsequently, he underwent other closure attempt and an unsuccessful enterocystoplasty that presented persisted dehiscent.

Patients were admitted to our institution at age of 13-year-old, and both had bladder and penile reconstruction unresolved.

**Figure 2:**
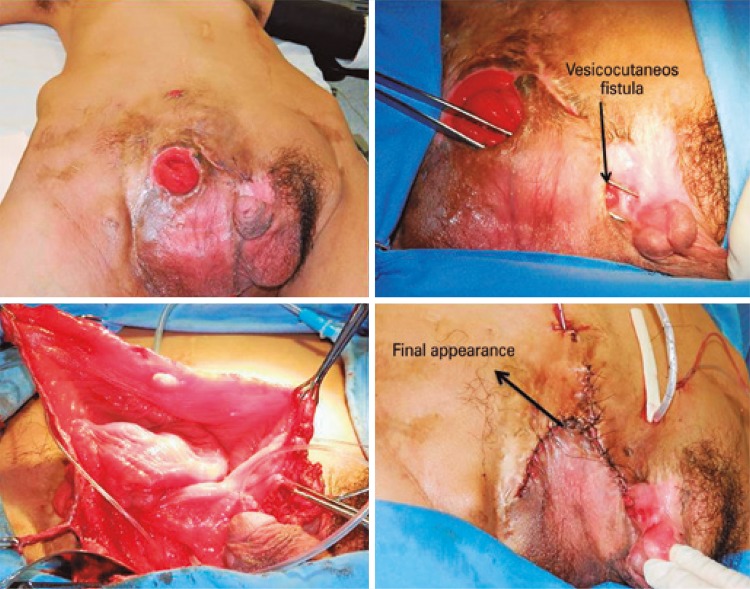
Visible neobladder in right lower abdominal quadrant, urinary stoma upwards, and open communication of bladder with penis

Upon clinical examination, twin 1 had visible neobladder at right lower abdominal quadrant, urinary stoma upwards and open communication of the bladder with the penis without surgical intervention ( Figure 2 ). Twin 2 did not undergo surgery, and he presented classical features of bladder exstrophy and epispadias. Patients’ main expectation was to achieve continence and abandon the use of diapers. We decided to assess intraoperatively the viability of previous enterocystoplasty underwent by twin 1, considering the possibility of closing bladder neck and repairing the efferent channel. For twin 2, we planned to create a continent catheterizable ileal reservoir as a pouch by using the concept developed by one of the authors of this report.^(^ ^4^ ^)^

**Figure 3:**
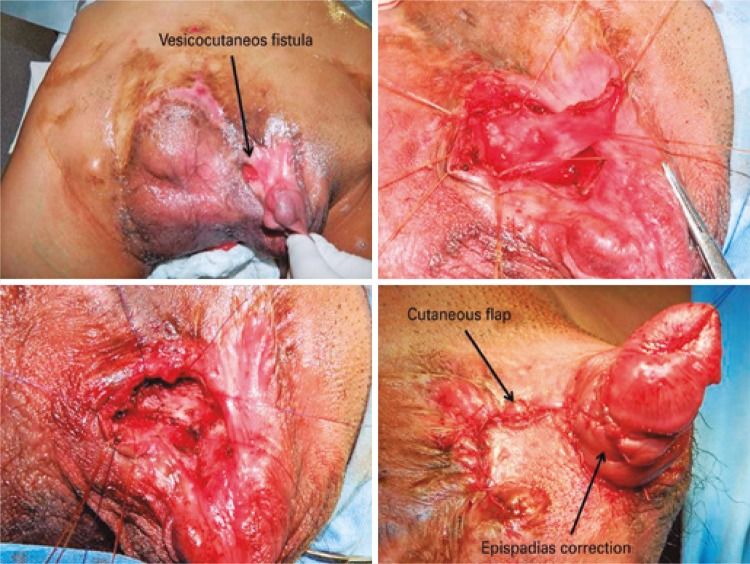
Fistula closure and epispadia repair

**Figure 4:**
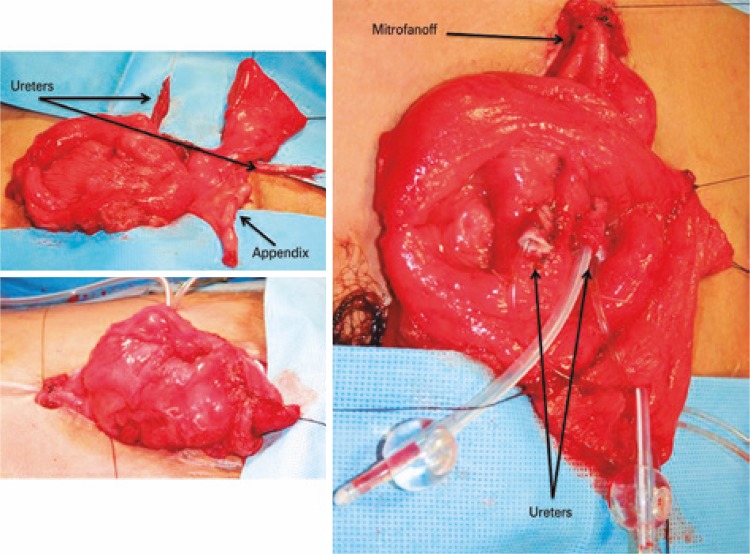
Construction of a pouch with additional ileum segment and use of appendix as outlet channel

After reassessing the neobladder of twin 1, we found a reasonable amount of tissue from the previous enterocystoplasty, a very long Monti tube displayed as a channel but anastomosed in the lowest part of the neobladder. We decided to reduce extension of the Monti tube in 50% and re-implanted it in the mid-part of the reservoir using the serous-lined concept.^(^ ^5^ ^)^ We closed bladder neck, neobladder and reconfigured the abdominal wall by rotating skin flaps to cover the defect ( Figure 2 ). The patient had a satisfactory outcome on abdominal wall, and bladder remained almost closed, but a fistula was developed in bladder neck area. After 6 months, the patient was underwent a new procedure to close the fistula and repair the epispadia ( Figure 3 ). Despite all efforts performed, a low flow fistula persisted in bladder neck area. For this reason, we decided to construct a pouch, discard the native bladder components and use only the part of intestinal neobladder. An additional ileal segment was needed and incorporated to the pouch for neobladder augment, ureters were implanted in the reservoir, and appendix was mobilized and used as an efferent channel ( Figure 4 ). The results was a patient with a continent reservoir and bladder emptying at 4 hour intervals.

**Figure 5:**
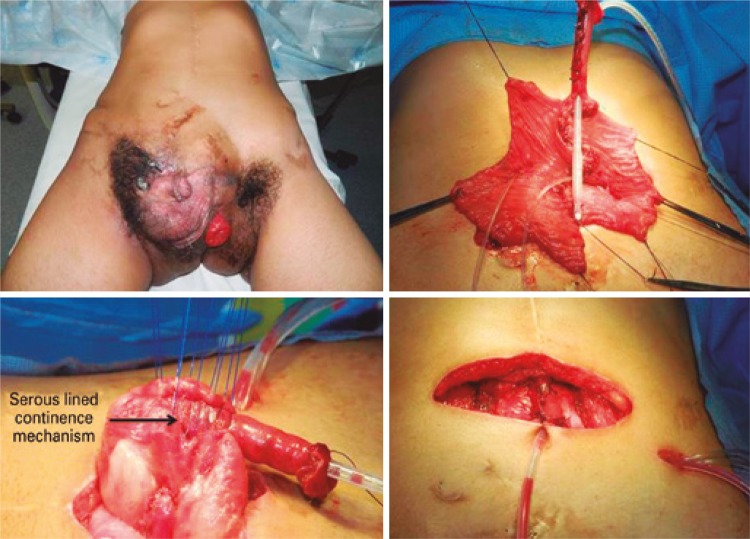
Immediately construction of a pouch using Macedo’s technique and discarding of native bladder

For the twin two, based on previous experience we had with his brother and the assumption that late primary bladder exstrophy closure was associated with higher incidence of dehiscence and fistula, we decided to construct immediately a catheterizable pouch 40cm from the ileum (Macedo’s technique). Ureters were implanted into the reservoir, and bladder plate was left behind without be excised ( Figure 5 ). Patient developed without intercurrences and currently he is continent for 4 hour intervals between catheterization. The mean follow-up was 8 months.

## DISCUSSION

In 1955, Spencer performed the first separation of ischiopagus twins, but only one twin survived.^(^ ^6^ ^)^ Pelvic fusion, as seen among symmetrical and asymmetrical ischiopagus and pygopagus twins, results in significant genitourinary anomalies, and the incidence of shared pelvic organs is 15% for pygopagus and 51% for ischiopagus twins.^(^ ^1^ ^)^

Most ischiopagus twins have four kidneys and two bladders. Our patients, although having one bladder each, shared the same challenges of bladder and penile reconstruction that are observed in exstrophic cases. Different from the classic concept of early bladder plate closure, the specific characteristics of patients imposed us a second intervention.

Bladder neck repair remains one of the critical aspects of bladder exstrophy treatment.^(^ ^7^ ^,^ ^8^ ^)^ Twin 1 had dehiscent enterocystoplasty and our attempts to reconstruct the bladder neck failed due to persistent urinary fistula. In such cases, *i.e* ., unsolved bladder exstrophy at adulthood, to make a shortcut and go right away to construct a continent catheterizable pouch is advisable. The Mainz-pouch is an interesting alternative for such purpose,^(^ ^9^ ^)^ however, in our case, we opted to use the ileum and although we used catheterizable ileal reservoir for bladder augmentation, we gained experience with urinary pouch as bladder substitute for rhabdomyosarcoma using the Macedo’s technique.^(^ ^10^ ^)^

Continence of channels is also a critical aspect of catheterizable channel’s techniques. A study carried out in Indiana, with mean follow-up of 28 months, reported that 97.5% (194/195) of patients included in the study continued to use their Monti catheterizable channel for bladder drainage. Of 199 patients, 17 (8,5%) required bladder or channel revisions. Of these patients, authors related as primary indications elongation and angulation of the channel in “7” and deficient tunnel length in “8”. Sixteen patients (8%) had minor difficulty with catheterization. Only 4 of 115 patients (3.5%) had leakage from the channel.^(^ ^11^ ^,^ ^12^ ^)^

Time to complications after the procedure may vary. A study carried out by Leslie et al., suggested that although the initial peak was followed by a relatively stable complication-free period, late problems can be found in long-term follow-up. The authors reported need of surgical revision in 39% of patients, most importantly for stomal stenosis (18%) and need of bulking agents injection (8%) to treat urinary leakage from the urinary stoma.^(^ ^13^ ^)^

Our experience with the Macedo’s technique has shown channel leakage of 13.6%.^(^ ^14^ ^)^ The continence principle of channels in this concept is based on tube embedding over a serous-lined tunnel. We learned from previous experience that angulation of the tube over bladder dome plays a significant role to promote continence.^(^ ^15^ ^)^ To enhance resistance we sought for alternatives. We found the Yachia technique, rectal muscle crossing over the tube.^(^ ^16^ ^)^ After adding this principle to Macedo’s technique, we were able to improve our results of continence from 87 to 100% in primary cases.^(^ ^17^ ^,^ ^18^ ^)^ Subsequently, we confirmed clinical results by pressure profilometry of the channel that conduit’s distal segment pressure in the studied group – using the Yachia’s technique (mean of 72.9 and peak of 128.7cmH_2_O), and this was significantly higher (p<0.05) than conduit’s distal segment pressure in the control group − without Yachia’s techinque (mean of 48.3 and peak of 65.1cmH_2_O).^(^ ^19^ ^)^

## CONCLUSION

Complex urinary reconstruction can be also performed by ischiopagus twins and this allow them to achieve continence and may improve their self-esteem even in cases of difficult clinical situations.
